# Recent Advances in Liposomal-Based Anti-Inflammatory Therapy

**DOI:** 10.3390/pharmaceutics13071004

**Published:** 2021-07-01

**Authors:** Carla M. A. van Alem, Josbert M. Metselaar, Cees van Kooten, Joris I. Rotmans

**Affiliations:** 1 Department of Internal Medicine, Leiden University Medical Center, 2333 ZA Leiden, The Netherlands; c.m.a.van_alem@lumc.nl (C.M.A.v.A.); c.van_kooten@lumc.nl (C.v.K.); 2 Institute for Experimental Molecular Imaging, Faculty of Medicine, RWTH Aachen University, 52074 Aachen, Germany; jmetselaar@ukaachen.de

**Keywords:** liposomes, inflammation, immune system, anti-inflammatory, therapy, innovation, formulation, administration

## Abstract

Liposomes can be seen as ideal carriers for anti-inflammatory drugs as their ability to (passively) target sites of inflammation and release their content to inflammatory target cells enables them to increase local efficacy with only limited systemic exposure and adverse effects. Nonetheless, few liposomal formulations seem to reach the clinic. The current review provides an overview of the more recent innovations in liposomal treatment of rheumatoid arthritis, psoriasis, vascular inflammation, and transplantation. Cutting edge developments include the liposomal delivery of gene and RNA therapeutics and the use of hybrid systems where several liposomal bilayer features, or several drugs, are combined in a single formulation. The majority of the articles reviewed here focus on preclinical animal studies where proof-of-principle of an improved efficacy–safety ratio is observed when using liposomal formulations. A few clinical studies are included as well, which brings us to a discussion about the challenges of clinical translation of liposomal nanomedicines in the field of inflammatory diseases.

## 1. Introduction

The field of nanomedicine has been rapidly expanding over the past decades [1,2,3], and liposomes were the first to reach the clinical application stage. The parenteral liposomal drug products currently on the market focus on the treatment of cancer through local delivery of chemotherapeutic drugs [4], fungal infections through local delivery of the antifungal drug amphotericin B [5], or pain management through prolonged epidural delivery of morphine [6].

In these cases, liposomes provide an improvement as treatment with the unencapsulated drug requires high and frequent dosing to achieve sufficient target site concentrations, which often leads to high systemic exposure and off-target toxicity [7,8,9]. Indeed, liposomes have been frontrunners in the field of nanomedicine owing to their nontoxic nature being composed of natural phospholipids, their relatively high drug loading capacity, and their flexibility to be modified in terms of size, charge, and surface features to enable optimal target localization [10]. This arguably renders liposomes the most well-studied nanocarrier for systemic and local drug delivery. Due to this altered bioavailability, drug efficacy can be enhanced while off-target adverse effects can be limited. Numerous clinical trials have been conducted to translate promising liposomal drug formulations from bench to bedside. 

Upon systemic injection liposomes can accumulate at the pathological target site through either active or passive targeting, where active targeting refers to the grafting of ligands onto the surface of the liposome, which can selectively bind to cell-specific receptors [11], such as E-selectin, which is expressed in inflamed endothelium [12]. In passive targeting, liposomes accumulate by means of escaping leaky vasculature present in inflamed and neoplastic tissues, enabled by their prolonged residence time in the circulation (hence, often referred to as ‘long-circulating liposomes’) [13,14]. This phenomenon is also known as the ‘enhanced permeability and retention’ (EPR) effect [11]. 

Interestingly, upon extravasation and entering the inflamed target site, liposomes show a natural propensity to be taken up by the local mononuclear phagocytic system, which includes macrophages and dendritic cells [15]. These cells often play key roles in the process of inflammation, including antigen presentation, proinflammatory cytokine production, and the release of tissue-degrading enzymes [16]. 

In addition to systemic administration routes, liposomes have been extensively investigated as potential drug carriers for percutaneous or even transdermal drug delivery in inflammatory diseases such as psoriasis [17]. This field of application has always existed somewhat isolated from the research into systemic liposomal drug delivery, but notable progress has been made that is worth mentioning.

This review summarizes the developments over the past decade in the fields of formulation and application of liposomal drug formulations for the treatment of severe inflammatory diseases, excluding cancer and infectious diseases. Innovations in late-stage animal models and clinical research will be discussed and will be accompanied by an overview of current limitations in clinical translation, in the discussion. 

## 2. Innovations in Liposome Formulation

Conventional liposomes are a type of nanomedicine composed of self-assembling phospholipid bilayers [18], creating a spheroid vesicle ranging from 30 nanometers to several micrometers in diameter [19]. The aqueous interior is well suited to hold hydrophilic drugs, while the lipid bilayer can be loaded with hydrophobic drugs. Several structural modifications can be made to these conventional liposomes to alter their characteristics, allowing them to gain additional features. 

### 2.1. The Liposome Lipid Bilayer

Liposomes are composed of an inner aqueous compartment surrounded by one or more concentric lipid bilayers constituted by membrane-forming amphipathic lipids (phospho- and sphingolipids) of natural or synthetic origin [20,21]. Often, other bilayer constituents are present, such as cholesterol, to enhance in vivo performance. Both liposomal vesicle size and surface charge are key features determining the in vivo fate of liposomes, but the bilayer morphology (unilamellar or multilamellar) and membrane fluidity are also important, the latter influenced by the ratio of saturated versus unsaturated lipids in the bilayer [22]. 

During the past decades, a range of added lipid bilayer features have been investigated in order to design liposomes:

(a) Showing prolonged circulation in blood upon infusion (“long-circulating liposomes”) [23,24];
(b) Enabling specific target cell interaction with surface-grafted peptides such as antibodies (“immune liposomes”) [25,26];
(c) Interacting with and enclosing negatively charged biomolecules such as nucleic acids (“cationic liposomes”) [27,28];
(d) Penetrating through the stratum corneum in order to reach inflammatory lesions in the skin (“deformable liposomes”) [29,30];
(e) Allowing for triggered release in the target tissues, e.g., upon temperature or pH changes (“stimuli responsive liposomes”) [31,32].

**Figure 1:**
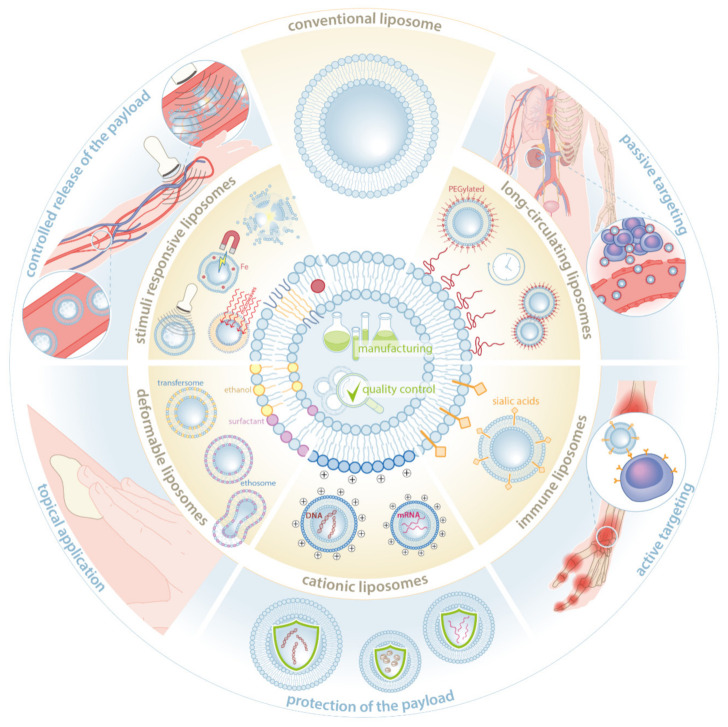
Summary of the features available for the modification of liposomes in order to provide beneficial clinical use. Five categories of liposome features currently available are presented in the middle ring of the figure, which include long-circulating liposomes, immune liposomes, cationic liposomes, deformable liposomes, and stimuli responsive liposomes. In the outer ring, the associated clinical benefits are illustrated, which include passive targeting, active targeting, protection of the payload, topical application, and controlled release of the payload, respectively. For targeting of specific pathologies or cells, several features may be combined to enhance clinical efficiency and/or safety in treatment of (severe) inflammatory diseases.

These properties are often strategically combined in one formulation. A graphical summary of these features and their benefits is presented in Figure 1.

Interestingly, unmodified conventional liposomes are already suited for passive targeting of inflammatory diseases, as they are internalized by inflammatory cells locally. Targeting and downregulating these cell types has been shown to be a rewarding strategy in many inflammatory disease models and also in patients [33,34].

Regarding the added liposomal bilayer features, (a) long circulating liposomes and (b) immune liposomes have especially been extensively researched in inflammatory disease. Indeed, long-circulating liposomes benefit from target tissue-selective accumulation at inflamed sites with enhanced vascular permeability (‘passive targeting’), while immune liposomes can be designed to selectively interact with activated endothelial cells or circulating immune cells in blood (‘active targeting’) [35,36].

More recent advances in terms of liposome lipid bilayer composition and surface features include specific formulations that are optimized for the encapsulation and delivery of plasmid DNA and RNA (notably mRNA and siRNA), creating liposomes that are also morphologically altered. Indeed, progress specifically in the field of inflammatory diseases has led to new therapeutic approaches, notably in arthritis and psoriasis [37,38]. 

Additionally, it is worth mentioning, specifically in inflammatory diseases, liposomes for the topical delivery of anti-inflammatory therapeutics on inflamed skin lesions with the aim to achieve transdermal delivery. Elastic liposomes (often referred to as ‘transfersomes’) and so-called ‘ethosomes’, which are conventional liposomal formulations featuring a significant percentage of ethanol, have been known for more than two decades, but recent improvements of these technologies have led to broadened applications in skin inflammatory diseases, and a wider range of drug molecules have now been investigated with these technologies [39,40]. 

### 2.2. Liposome Manufacturing and Quality Control

Liposomes can be made by dispersing phospholipids directly in an aqueous buffer or by first dissolving the phospholipids together with the other lipophilic constituents in an organic solvent, transferring it to a glass container from which the solvent is later evaporated. The resulting dry lipid film is then shaken with the aqueous buffer to disperse the lipids (referred to as the ‘lipid film hydration’ method) [41]. Alternatively, the dissolved lipid mixture is directly injected into the aqueous dispersion buffer, a process that is only possible when the organic solvent is miscible with water [42]. Most liposome formulations involve a sizing procedure that refines the crude formulation and turns it into a smaller, more uniformly distributed vesicle dispersion that is critical if the liposomal product is to be injected for systemic targeting of sites of inflammation [43]. 

Sizing is traditionally done by high-sheer homogenization or extrusion through polycarbonate membrane filters and typically yields liposomes of around 0.2 μm or smaller (referred to as ‘small unilamellar vesicles’ or SUVs). More recent and advanced liposome manufacturing technologies often feature so-called microfluidic chips in which the whole process of mixing and sizing is achieved in one controlled mixing step using (relative) flow speeds, temperature, and pressure as the critical process parameters that control the ultimate vesicle size [44]. Dialysis or ultrafiltration are implemented to clear the liposomal dispersion of the remaining solvent and the unincorporated water-soluble actives or excipients, while dead-end 0.2 μm filtration ensures sterility upon filling the dispersion into containers or vials [43].

An interesting development partly triggered by the exploration of micro- and millifluidics in the production of liposomal and other nanomedicine products is continuous flow manufacturing [45,46]. As opposed to conventional batch production, continuous flow manufacturing allows better control over particle size and drug loading during manufacturing by measuring these characteristics in-line while the mixing and sizing process is running. This allows the operator to immediately change the critical process parameters if the specifications are not met, reducing the chance of eventual batch failures. Furthermore, scaling up is becoming less risky since the only variable that now determines the final volume produced is the overall process duration. One could even envisage this method in a personalized medicine setting: e.g., when an individual patient’s biomarkers point to a specific set of cell receptors being upregulated at their inflamed lesions, these could be targeted by a bespoke immune liposome formulated and produced under clinical conditions on the spot right before treatment.

To ensure robust in vivo performance, batches of liposomal drug products need to remain within a list of tightly defined specifications, which typically include the following: liposome vesicle size and polydispersity index, drug and lipid excipient content (including drug to lipid ratio), pH, osmolality, and drug retention/release rate. Limits will have to be set to the unencapsulated drug content, residual solvent, microbial contamination (parenteral liposomal drug products need to be sterile), and endotoxin content [47,48].

Particle size and polydispersity index are typically measured by dynamic light scattering (DLS), but other methods have recently become more widely used, such as nanoparticle tracking analysis (NTA). Drug and lipid excipient content are typically measured with HPLC using UV detection. However, many lipids can be better detected with refractive index (RI), evaporative light scattering (ELSD), or charged aerosol detection (CAD). Adequate methods must be found to separate the unencapsulated from encapsulated drug, possibly by centrifugal membrane concentrators or size exclusion columns. Notably the degree of drug retention and/or release must be gauged, which is typically done with an in vitro release assay under forced conditions [49,50].

An important aspect of liposomal drug product development is the stability on storage. Liposomal products are often stored between 2 and 8 degrees Celsius to maximize storage duration. Freezing is in principle to be avoided due to the potentially detrimental effects of ice crystals on the delicate phospholipid vesicle structure. Nonetheless, in some cases, freezing is possible if a carefully selected cryoprotectant is included in the formulation, of which trehalose is a prime example. With some liposome formulations, this excipient may even allow freeze drying, which has the additional advantage of allowing (water-free) storage at room temperature [51,52].

## 3. Application in Inflammatory Disease

**Figure 2:**
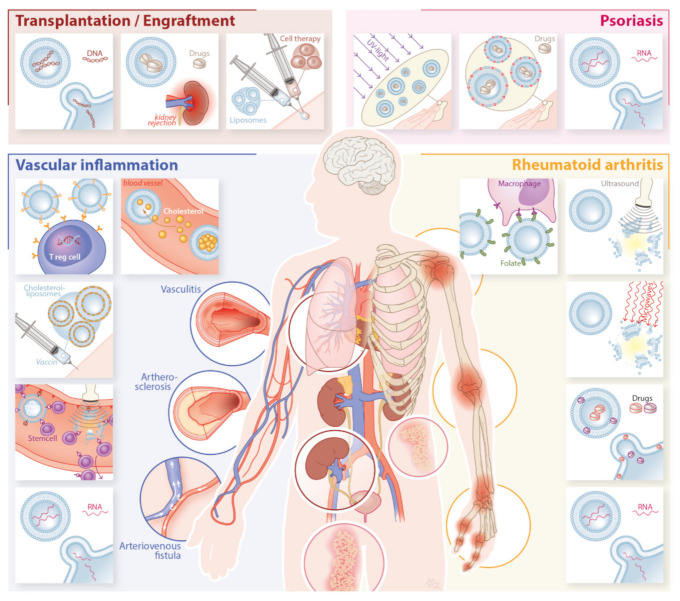
Summary of the innovative developments in liposomal treatment of the inflammatory conditions psoriasis, rheumatoid arthritis, vascular inflammation, and transplantation and engraftment. Innovations are presented per field and include developments in topical applications, RNA drug delivery, and PUVA therapy in psoriasis; codelivery of drugs and heat-responsive drug release in rheumatoid arthritis; cholesterol vaccination, induction of Tregs, and ultrasound-mediated drug release in vascular inflammation; and treatment of acute graft rejection in transplantation.

Inflammation is involved in a variety of different pathologies, including infections, autoimmune diseases, cancer, and rejection of transplant organs. Several vascular effects occur at the site of inflammation, including locally enhanced vascular permeability [53]. At these sites, where the endothelial barrier is more porous, fluid, proteins, and even cells (notably leukocytes) can diffuse from the vasculature into the interstitial space [54]. It is this mechanism which allows liposomes to selectively accumulate in inflamed sites (referred to as passive targeting), as they cannot cross the healthy endothelial barrier but only extravasate into inflamed tissues. Additionally, during inflammation, antigen presenting cells such as macrophages are involved from the (acute) injury until the repair and regeneration phase [55]. These cells have a phagocytic function and have been shown in vitro to internalize liposomes within 8 h [56]. Combined, the local extravasation and subsequent cellular uptake of liposomes make inflammatory disease an ideal target for passive liposomal drug delivery. Indeed, a great number of preclinical studies have been conducted to assess whether liposomal drug delivery is beneficial in the treatment of specific inflammatory conditions which have been summarized in Figure 2.

### 3.1. Rheumatoid Arthritis

Rheumatoid Arthritis (RA) is a chronic disease where inflammation is triggered by an autoimmune response [57]. RA initially affects smaller joints but can progress to affect internal organs and the skin as well [58]. It is a highly prevalent condition, with 0.3–1% of the world’s population affected [59]. With no cure, treatment of the disease is focused on alleviating symptoms using anti-inflammatory drugs, biologicals, disease modifying anti-rheumatic drugs, or even nucleic acid-based drugs [58,60]. In order to improve targeted delivery to affected joints, intra-articular treatment through local injection is often employed, but due to the need for injection of all affected joints, this is a suboptimal solution. As liposomes can be modified to be taken up by specific cell types and can be modified to enhance and prolong retention, the treatment of RA with liposomal drugs is studied pre-clinically and in clinical trials.

The anti-inflammatory drugs used to treat RA can be classified as non-steroidal anti-inflammatory drugs (NSAIDs) and glucocorticoids [61]. NSAIDs are used primarily to manage pain and are taken orally, and the most common adverse effects include abdominal pain, diarrhea, and nausea, while serious side effects such as hypertension and kidney failure can also occur [62]. Targeted delivery of NSAIDs such as diclofenac in RA has not been studied in clinical trials, but preclinical studies have shown promising results in rat models of arthritis [61]. Glucocorticoids exert their effect through binding to the glucocorticoid receptor and inducing the downstream regulation of (anti-) inflammatory processes [63]. More than 50% of RA patients use glucocorticoids as they are very effective in alleviating RA disease activity [64], but long-term use leads to the onset of harmful side effects such as osteoporosis, hypertension, diabetes mellitus, and increased susceptibility to infections [65,66]. They can be administered orally, intramuscularly, intravenously, or via intra-articular injection [63]. Targeted delivery using liposomes has most frequently been studied with prednisolone or dexamethasone, and preclinical studies show increased efficacy of these drugs [67,68,69].

Disease modifying antirheumatic drugs (DMARDs) all have a unique mode of action, but many of these also show adverse effects [70], including an increased susceptibility to viral and bacterial infections. Well-known DMARDs include (I) methotrexate, which is a potent inhibitor of pro-inflammatory cytokines IL-1, IL-6, and IL-8, (II); sulfasalazine, which inhibits chemotaxis and migration of inflammatory cells [71]; (III) clodronate, which selectively depletes macrophages in a liposomal form [72], (IV) hydroxychloroquine, which inhibits Toll-like receptor 9; (V) tofacitinib, which interferes with cytokine signaling through JAK inhibition [73,74,75]; and (VI) leflunomide, which inhibits pyrimidine synthesis [76,77]. Clodronate- and methotrexate-containing liposomes have especially been well studied in RA [78,79], with clodronate liposomes having been evaluated in a clinical study. In this study, patients scheduled for a knee replacement surgery received an intra-articular injection with clodronate liposomes a week before surgery and underwent a synovial tissue biopsy at 2 weeks prior and at the time of surgery. The presence of synovial macrophages was significantly reduced at the time of surgery, and the procedure was well tolerated [34]. In a critical note by the authors, they indicate that although macrophage presence is correlated with damage, such damage is accumulated over years of chronic inflammation, and macrophage activity might fluctuate in this period. Clinical efficacy studies are planned.

Biologicals for the treatment of RA form a subclass of DMARDs and anti-inflammatory drugs, are administered subcutaneously and intravenously, and include monoclonal antibodies, which inhibit production of pro-inflammatory cytokines, such as IL-1, IL-6, and TNF, or directly inhibit B-cells [61]. Additionally, with these treatments, adverse effects are related to systemic immunosuppression and include opportunistic viral and bacterial infections. As this form of RA treatment is already a type of targeted treatment, liposomal encapsulation cannot be expected to add much in terms of target localization and efficacy.

Nucleic acid-based drugs (NABDs) are currently being investigated for use in RA treatment. No product is on the market yet. Since the exact cause of RA is still unknown, a single curative NABD therapy cannot be designed. However, as RA is known as an auto-immune disorder, NABDs can be designed to target inflammatory factors involved in the propagation of the disease. Potential targets include matrix degradation enzymes, pro-inflammatory cytokines, and microRNAs which regulate gene expression [80]. One of the main challenges in the delivery of NABDs is their rapid degradation and clearance, which can be remedied by liposomal encapsulation [81].

The use of conventional drugs in liposomal form for the treatment of RA has been reviewed extensively [61,82,83]. Nearly all studies discussed in these reviews reported increased efficacy or localization of the liposomal drug when compared to conventional treatment [61,83,84]. The overview in Table 1 provides a summary of innovative research conducted in this field. Recent notable innovations include liposomal drugs where liposomes are modified for local accumulation via peptide binding or folate conjugation or modified for local release through reactivity to pH, ultrasound, or thermosensitivity [85,86,87,88]. Additionally, conventional drugs are combined and encapsulated in liposomes to enable codelivery in order to evaluate a potential synergistic effect [89,90,91,92]. Additionally, drugs that have been abandoned due to extensive side effects have been re-evaluated in a liposomal form. Liposomal administration of a sulfapyridine prodrug led to better intra-articular retention and increased therapeutic efficacy [93]. Some of these short term studies even indicate a decrease of adverse effects [94], which is noteworthy as adverse effects are generally detected only after sustained exposure in longer term studies.

### 3.2. Psoriasis

Psoriasis is an autoimmune disorder that primarily affects the skin and appears as red patches or scaly plaques that cause itching. However, the inflammatory reaction can also progress to involve the joints [111]. Psoriasis occurs in approximately 2% of the population [112], although it has a geographical variance, and is associated with severe co-morbidities such as metabolic syndrome and cardiovascular diseases, which further increase the burden of disease for these patients [111,113]. Although the exact cause of this multifactorial disease is unknown, research has shown that mediators of the adaptive immune system are integral in disease progression [114]. The so-called TNF-α/IL-23/IL-17 axis is initiated by activated plasmacytoid dendritic cells which produce TNF-α, which in turn results in IL-23 production by recruited inflammatory dendritic cells. IL-23 then activates T helper type 17 cells which produce IL-17, leading to the proliferation of keratinocytes [113]. Psoriasis can manifest itself in different types, of which chronic plaque psoriasis is the most common form [111]. Plaque psoriasis treatment is dependent on the severity of the disease. When less than 5% of the body surface area is affected, topical treatment or targeted phototherapy is used. When more than 5% of body surface area is involved, systemic treatment with oral medication, biologics, and/or phototherapy are employed [115].

Topical treatment for psoriasis includes topical corticosteroids, vitamin B analogues, calcineurin inhibitors, and keratolytics [115]. Targeted phototherapy is also available but is excluded for the purposes of this review. Adverse effects are not highly frequent using topical applications, but the use of topical corticosteroids has been associated with well-known corticosteroid-related systemic adverse effects as well as local side effects, including skin atrophy, telangiectasia, and striae. Use of topical calcineurin inhibitors, however, can increase the risk of malignancies.

Oral treatments include (I) Methotrexate, which inhibits cell growth and decreases scale forming in psoriasis but also leads to adverse effects, such as gastrointestinal complaints, bone marrow suppression, and liver function abnormalities [116]; (II) Apremilast, which is a DMARD and is often prescribed to patients who also suffer from psoriatic arthritis. It is effective in treating moderate to severe plaque psoriasis, and adverse effects are relatively mild and include nausea, headache, and upper respiratory tract infection [117]; (III) Acitretin, which is an anti-inflammatory and antiproliferative drug which is mostly used in combination with phototherapy to increase its efficacy. Adverse effects occur mainly in high dosages and include teratogenicity, mucocutanous toxicity, skeletal toxicity, idiopathic intracranial hypertension, myalgias, and arthralgias [118]; (IV) cyclosporine is a calcineurin inhibitor which inhibits IL-2 transcription and thereby negatively regulates T-cell activation [119]. Adverse effects include nephrotoxicity, hepatotoxicity, and electrolyte elevations [120]; and (V) PUVA (psoralen and UV-A), a highly effective therapy, which is a combination of an oral psoralen with UV-A phototherapy thought to work synergistically but is limited due to the onset of severe adverse effects such as squamous cell carcinoma [121].

Systemic treatment of psoriasis often involves biologics such as anti-TNF-α, anti-IL-17, anti-IL-12/23, and anti-IL-23 monoclonal antibodies. As these drugs all interfere with the adaptive immune system, side effects are linked to immunosuppression, and opportunistic infections such as mucocutaneous candidiasis can occur. In rare cases, malignancies such as basal cell carcinoma have been reported [115].

For several of the drugs used in the treatment of psoriasis, the adverse effects are so severe that some form of locally enhanced delivery is warranted, and liposomal encapsulation seems to be an obvious choice. Other drugs face challenges in terms of percutaneous permeability, something which can also potentially be improved with liposomal encapsulation. Innovations in this field include novel combination therapies with liposomal drugs and ultraviolet light where adaptations to the liposome composition, such as charge adaptation, lead to increased dermal penetration, allowing for topical PUVA therapy, which reduced psoriasis symptoms in a psoriatic plaque model [122]. A comparable study was performed for the topical delivery of cyclosporine, where dermal absorption is also a limiting factor. Here, adapting the liposome size, adapting the zeta potential, and increasing the membrane flexibility to further permeate the skin resulted in an increased efficacy of topical cyclosporine [123]. The application of topical cyclosporine was also evaluated clinically, where treatment with cyclosporine lipogel resulted in complete clearance in 41% of psoriasis lesional sites in a safe manner [124]. Even though liposomal formulations often result in an improved efficacy of the encapsulated drug, other formulations may be more suited, as was revealed in a clinical study where anthralin was loaded into a liposomal and ethosomal gel. In a clinical study of psoriasis patients using either drug formulation, the efficacy of the ethosomal gel exceeded that of the liposomal formulation [125]. Another area of innovation is the liposomal encapsulation of biologics, where liposomal spherical nucleic acids target the IL-17 receptor. This formulation was evaluated in a mouse model of imiquimod-induced psoriatic plaque and resulted in the reversal of psoriasis symptoms [38]. Some of these innovations have also been reviewed earlier by several groups [17,126,127,128], and an extended overview is presented in Table 2. Promising results are obtained with a combination therapy of liposomal drug release using laser irradiation, where no recurrence of symptoms was observed in treated mice [129]. Additionally, the encapsulation of dithranol in liposomes resulted in a promising outcome, as five out of nine patients treated were totally cleared of lesions, and a 50% reduction was achieved in two other patients [130].

### 3.3. Vascular Inflammation

Vascular inflammation occurs in several pathologies, such as vasculitis, atherosclerosis, and arteriovenous fistula formation [138,139,140]. 

Vasculitis can present itself in various forms, such as giant cell arteritis, antineutrophil cytoplasmic antibodies (ANCA) vasculitis, and up to 30 more specific pathologies [141]. A central factor is vessel inflammation and activation of autoantigen-specific T cell immunity. The first-line treatment for vasculitis is a combination of glucocorticoids and an immunosuppressive agent, such as methotrexate, azathioprine, mycophenolate mofetil, cyclophosphamide, or rituximab, depending on the type of vasculitis [138]. An innovative approach to treat vasculitis has been described by Galea et al., who used a mouse model of Goodpasture’s vasculitis treated with liposomes containing calcitriol and an antigenic peptide to induce tolerance in dendritic cells and T-cells. To achieve tolerance, liposomes were targeted to PD-L1^hi^ dendritic cells in draining lymph nodes where expression of the MHC-class II receptor was downregulated, leading to both reduced function of effector T-cells and promotion of regulatory T-cells [142]. The efficacy of liposomal formulations containing corticosteroids has not been evaluated in vasculitis.

Atherosclerosis onset is mediated by monocytic influx into the vessel wall where lipid uptake leads to the formation of foam cells. These lesions form into plaques after infiltration of smooth muscle cells, and unrestricted growth can lead to blockage of the blood vessel. Treatment of atherosclerosis depends on the severity and location of the condition and can consist of medical intervention to decrease circulating lipids [143] or surgical intervention, such as stenting or angioplasty [144]. Liposomes can be used to directly lower cholesterol in the circulation, which is a major risk factor in atherosclerosis [143], through cholesterol entrapment. In an animal study with cholesterol-fed rabbits, weekly infusions with dimyristoylphosphatidylcholine (DMPC) liposomes resulted in a significantly decreased aortic cholesterol content and decreased atherosclerotic plaque volume [145]. Alternatively, liposomes can be used to vaccinate against cholesterol, by inducing anticholesterol IgG and IgM antibodies through injection of rabbits using liposomes containing 71% cholesterol and lipid A as an adjuvant. This strategy resulted in a decreased elevation of serum cholesterol accompanied by reduced antibody levels, indicating an antibody-mediated decrease. A decreased artherosclerosis risk and decreased plaque size were also observed [146]. Apart from direct interference with cholesterol metabolism, liposomes are also utilized in this field to locally deliver drugs, and a rabbit model with artherosclerotic plaques induced by a high cholesterol diet was treated intravenously with prednisolone phosphate in PEGylated liposomes. Local accumulation in the plaque was achieved, and prednisolone treatment was efficient [147]. However, in a clinical study of patients with atherosclerotic disease, intravenous injection with liposomal prednisolone led to an accumulation in plaque macrophages, but anti-inflammatory efficacy was not observed [148]. In a mouse model using ApoE knockout mice, a statin was delivered using a high-density lipoprotein nanoparticle which resulted in a decrease in atherosclerotic plaque inflammation progression [149]. Furthermore, liposomes are used in RNA based drugs. For example, ApoE-deficient mice were injected with fatty acid-binding protein 4 (FABP4) si-RNA in liposomes, which resulted in successful delivery of siRNA to atherosclerotic plaques and successful suppression of FABP4 expression [150]. Additionally, stem cell therapy, potentially through ultrasound-mediated release, is delivered via liposomes, which was studied in an animal model. Pigs on a high cholesterol diet were treated with CD34 and ICAM-1 coupled echogenic immunoliposomes, and the associated stem cells were successfully delivered to the arterial intima, which was enhanced upon ultrasound treatment [151]. 

Arteriovenous fistula (AVF) is the preferred mechanism for vascular access in hemodialysis patients. They are surgically created but often fail to mature [152]. The exact mechanism of maturation failure is unknown, but research has shown that impaired outward remodeling and increased intimal hyperplasia both play an important role in AVF nonmaturation [153]. In these processes, infiltration of inflammatory cells was observed [153,154,155]. The standard-of-care is a percutaneous transluminal angioplasty where the fistula is surgically reopened. Recently a liposomal formulation of prednisolone was evaluated in a mouse model of AVF in which vascular inflammation was inhibited, and outward remodeling was enhanced [156]. In a randomized clinical trial, liposomal prednisolone was determined to be safe for administration in patients after AVF surgery, but an increase in successful AVF maturation was not observed. 

An overview of these innovations is presented in Table 3.

### 3.4. Solid Organ Transplantation

Organ transplantation is the preferred treatment option for patients with end stage organ diseases [168]. In order to prevent rejection of the transplanted organ, recipients are treated using immunosuppressive medication. The treatment strategy usually includes an induction therapy with a monoclonal antibody at the time of transplantation [169], although this may vary per organ type [170,171], followed by a daily regimen of calcineurin inhibitors, glucocorticoids, and mycophenolate mofetil, or a combination of these [172,173]. Nevertheless, rejection still occurs in 10% of all patients [174], which is treated with high-dose corticosteroids in the case of cellular rejection [175] and plasmapheresis, intravenous immunoglobulin, or T- or B-cell depleting agents in the case of antibody-mediated rejection [176]. Both the acute and chronic drug treatments are associated with severe adverse effects, such as the onset of malignancies and infections [177]. 

Liposomes have been explored in this field for quite some time in order to reduce adverse effects and increase drug efficacy. This started with liposomal encapsulation of standard-of-care drugs such as cyclosporine and methylprednisolone, where increased efficacy was reported in rat models of liver, cardiac, and renal transplantation, respectively [178,179,180]. Innovations in the field of liposomal therapy include gene therapy to increase graft survival, targeted drug delivery to treat rejection, and a combination of liposomal immunosuppressants to increase cell engraftment. These treatment options can target several different aspects of transplantation, as inflammatory responses are involved at multiple timepoints during transplantation. 

Immediately following transplantation, ischemia reperfusion injury occurs. Despite many interventional studies, no golden standard treatment for ischemia reperfusion injury is available. In a preclinical study using a kidney IRI model in rats, liposomes were shown to passively target the inflamed kidney [56]. Current innovation is focused on hypo- and normotherm machine perfusion to prevent ischemic injury [181], which provides an opportune window for additional interventions such as RNA interfering drugs [182]. In an ex vivo machine perfusion setting, the administration of lipid-coated siRNA molecules to a human liver resulted in uptake of these drugs by hepatocytes [183]. Next, as the recognition of non-self upon engraftment can be an initiator of the destructive immune response, studies have also been conducted to codeliver immunosuppressive drugs during cell grafting as a preventative strategy, where engraftment and survival was increased [184]. When acute rejection does occur, extreme doses of prednisolone are utilized as treatment, which in turn can induce (chronic) conditions such as diabetes mellitus, osteoporosis, and hypertension [185]. This particular condition is therefore well suited to target using liposomes in order to minimize systemic exposure. In an initial efficacy study in a mouse kidney transplantation model, the localization of liposomal prednisolone to the inflamed kidney was increased, and the efficacy of liposomal prednisolone in treating acute rejection was enhanced [186]. Other innovations in the field focus on gene therapy, where a study of cardiac transplant in a rabbit model was conducted to study the liposomal gene transfer of IL-10. Here, the absolute success of gene transfer was lower compared to adenovirus-based transfer, but the efficacy in terms of graft survival was higher [187]. In 2019, a novel liposomal delivery mechanism in the field of transplantation was studied clinically, where inhaled liposomal cyclosporine was used in the treatment of bronchiolitis obliterans syndrome post lung transplantation. Here, an increased treatment efficacy was observed while no systemic toxicity was reported [188].

An overview of the innovations in this field is presented in Table 4.

## 4. Discussion

In recent years, the development of liposomal formulations has shifted from mere encapsulation of existing small-molecular therapeutic agents to using liposomes as a technology platform to create truly novel therapies, such as cholesterol vaccines [158] and targeted delivery of RNA-based therapeutics, with encouraging preclinical results [38,90]. Reports from preclinical studies show positive results in terms of targeting and efficacy, where liposomal formulations are often compared to drugs in their free form, indicating superior efficacy over unencapsulated drugs. Although the use of liposomes in inflammatory disease has been—and still is—intensely studied up to the preclinical proof-of-principle, relatively few of these innovations reach clinical stage. The lack of translation can be attributed to several limiting factors. 

In the scientific aspect, results from preclinical animal models often do not translate to the clinical situation very well. This may be due to differences in in vivo performance of drugs and nanocarriers between animals and humans, which is already described for glucocorticoids that are extensively used in the treatment of inflammatory diseases [192,193]. Additionally, animal models often do not reflect the more complex system of human inflammatory pathology as, e.g., the APOE knockout mice studied in atherosclerosis lead to a rapid onset of atherosclerosis compared to human pathology and poorly reflected the polymorphism of the human APOE gene, leading to six different genotypes [194]. Furthermore, patients suffering from severe inflammatory disease often suffer from comorbidities with an inflammatory aspect, which is not reflected in animal models. Atherosclerosis is often seen as a comorbidity in patients suffering from chronic (inflammatory) diseases such as diabetes mellitus and rheumatoid arthritis [195], which may significantly impact the distribution of liposomes, leading to suboptimal dosing at the target site in a clinical setting. In the case of clinical research, the trials may fail due to, for example, suboptimal inclusion rates, limited statistical power, or selecting the wrong outcome measure [196]. 

Alternatively, there is also a business case to consider in developing a novel drug (formulation), which may increase the hurdle of reaching the clinic. As drug development is costly and full of risks, it is a challenge to secure sufficient funding, be it from grants, investors, or other sources [197,198]. Apart from the preclinical results, there are additional factors which can affect the attractiveness of the business case. The total available market, the time needed to reach the market, the financial planning, and, of course, the team are all factors evaluated by funding agencies prior to providing the funds required for clinical trials. Although these challenges come with all business propositions in drug development, the fields of nanomedicines and liposomal formulations face additional challenges related to the mechanism of action, product manufacturing, and quality control. Targeted drug delivery using nanomedicines significantly alters the biodistribution of the drugs to increase efficacy. However, this intended redistribution of the drug often also entails enhanced exposures to certain nontarget organs, most notably secondary lymphoid organs in the case of the systemic administration of nanomedicine products. Furthermore, the toxicology of the excipients and nanocarriers themselves have not always been fully explored in humans, something which can lead to unexpected toxicity issues, a notable example being the nanocarrier-induced hypersensitivity reactions leading to the activation of the complement-cascade that were not seen in rodents [199]. Therefore, additional preclinical safety studies, both in vitro and in vivo in relevant nonrodent animals, are recommended [200]. Following safety studies, the efficacy of a liposomal drug product can be assessed in a superiority design, or, as major benefits for liposomal application are associated with the reduction of adverse effects, the trial can be designed to show the reduction of adverse effects through a noninferiority design. In both designs, large study populations are required. To improve patient selection during clinical trial enrolment and in commercial application, companion diagnostics using biomarkers are often developed to preselect eligible patients [201]. In addition to the assessment of safety and therapeutic efficacy, an additional challenge arises in the quality control of these novel products, as not only does the active pharmaceutical ingredient need to be assessed, but additional assays are also needed to study the liposome quality. Additionally, challenges can arise in the long-term storage of liposomal formulations, as these products are prone to damage induced by oxidation hydrolysis [202]. Finally, the required route of administration of these products can form another hurdle, as nonliposomal counterparts of liposomal drug products are often available in a pill, while liposomal drug products often require I.V. administration, which may deter end users (clinicians and patients) in adopting such new therapies. Despite these significant challenges, several clinical trials have been conducted where liposomal drug formulations have been positively evaluated for treatment of inflammatory disease, such as rheumatoid arthritis, psoriasis, atherosclerosis, and transplantation [124,166,188,203], but not all trials result in progression to publication and/or the clinic [203]. 

For inspiration to foster further innovation in the field and to overcome these challenges, we can look to other fields of application. In oncology, several liposomal drugs are already on the market [204], and innovations such as light-induced cargo release are at the forefront of technological development [205]. Especially in the sector of cancer immunotherapy, innovations can have an overlapping application with anti-inflammatory targets [9]. An illustrative example of overlap in these two fields is the development of liposomes targeted to PD-L1, as several therapies directed at PD-L1 are in clinical use and clinical development for the treatment of several cancer types [206,207]. Simultaneously, PD-L1 is also targeted in the treatment of vasculitis and rheumatoid arthritis in preclinical research [142]. 

In addition to inspiration from other fields of application such as oncology, innovations within the field of anti-inflammatory therapy can be transferred to other target conditions. In the most prominent anti-inflammatory disease targeted by liposomal drug development, rheumatoid arthritis, innovation is mostly focused on combining several therapies [89,90]. In the other anti-inflammatory disease applications discussed in the current review, such as psoriasis, liposomal formulations are now also considered for combination therapy [133]. This type of knowledge transfer is essential for the development of novel therapeutic options in all fields of application.

Following the scientific and technological innovation in the field, an optimal combination of positive preclinical data, a strong business case, and an excellent team should bring these innovations from bench to bedside.

## Figures and Tables

**Table 1:** Overview of innovative research using liposome-based drug delivery in the treatment of RA.

Drug Class	Drug	Animal	Model	Route	Effect *	Ref.
Glucocorticoid	Prednisolone phosphate in PEGylated liposomes	Mouse	Antigen-induced arthritis	I.V. *	Effective inhibition of inflammation and potential reduced bone erosion	[94]
Glucocorticoid	Dexamethasone phosphate in liposomes	Mouse	Collagen-induced arthritis	I.V.	Persistent anti-inflammatory effect, limitation of the suppression of the HPA axis, absence of the drug-induced gluconeogenesis	[95]
Glucocorticoid	Methyl prednisolone hemisuccinate in pegylated nanoliposomes	Rat and Beagle	Adjuvant-induced arthritis	I.V.	Superior therapeutic efficacy to free glucocorticoid	[96]
DMARD *	Prodrug of sulfapyridine in liposomes	Rat	Adjuvant-induced arthritis	I.A.*	Reverse the symptoms ofinflammation	[93]
DMARD	Sinomenine hydrochloride in thermosensitive liposomes	Rat	Adjuvant-induced arthritis	I.V.	Superior antirheumatoid arthritis effect	[85]
DMARD	Methotrexate and indocyanine green loaded iRGD peptide-functionalized echogenic liposomes	Mouse	Collagen-induced arthritis	I.V.	Greatly improved therapeutic efficacy and reduced methotrexate side effects, allows for ultrasound mediated release	[87]
DMARD	Dimeric artesunate phospholipid conjugate in liposomes	Rat	Adjuvant-induced arthritis	I.V.	Significantly higher inhibition of the cell secretion of proinflammatory cytokines	[91]
NABD *	TNF-α small interfering RNA wrapsome vs. control si-RNA	Mouse	Collagen-induced arthritis	I.V.	Significant decreases in severity of arthritis and TNF-α mRNA	[97]
NABD	anti-IL-1, anti-IL-6, or anti-IL-18 small interfering RNA in lipoplexes vs. anti-TNF siRNA lipoplex-based treatment	Mouse	Collagen-induced arthritis	I.V.	Significantly reduced the incidence and severity of arthritis	[98]
NABD	TNF-α small interfering RNA in a cationic liposome formulation vs. untreated	Mouse	Collagen-induced arthritis	I.P.*	Inhibition (50–70%) of articular and systemic TNF secretion	[99]
Hybrid	Dexamethasone sodium phosphate in Folate conjugated PEG liposomes	Rat	Collagen-induced arthritis	I.V.	Improved drug release under ultrasound vs. unstimulated liposomes	[100]
Hybrid	Prednisolone and Methotrexate in PEGylated liposomes	Rat	Carrageenan induced arthritis	I.V.	Higher inhibition of edema and increased local accumulation of double liposomes compared to single layer liposomes	[89]
Hybrid	Prednisolone phosphate and Fludeoxyglucose (18 F) in PEGylated liposomes vs. untreated	Mouse	Antigen-induced arthritis	I.V.	Increased uptake in inflamed joints, suppression of joint swelling after treatment	[101]
Hybrid	Dexamethasone in Sialic Acid Conjugate–Modified Liposomes in a size range	Rat	Adjuvant-induced arthritis	I.V.	Neutrophil targeting was achieved with small liposomes, anti-RA efficacy was established	[102]
Hybrid	Dexamethasone palmitate in Sialic Acid Conjugate–Modified Liposomes	Rat	Adjuvant-induced arthritis	I.V.	Accumulation in peripheral blood neutrophils, strong anti-inflammatory effect	[103]
Hybrid	Dexamethasone in liposomes with a novel peptide ligand ART-2	Rat	Antigen-induced arthritis	S.C. *	Enhanced endothelial cell-binding, increased efficacy compared to free drugs	[104]
Hybrid	Betamethasone in folate-conjugated liposomes vs. untargeted liposomes	Rat	Adjuvant-induced arthritis	I.V.	Less paw swelling, lower arthritis scores, a reduction in bone erosion, less splenomegaly and better maintenance of body weight	[105]
Hybrid	Prednisolone in peptide targeted liposomes	Rat	Adjuvant-induced arthritis	I.V.	Local accumulation vs. unaffected joints, and minimal inflammation in vivo	[106]
Hybrid	Methotrexate and catalase co-encapsulated in folate and PEG conjugated liposomes	Mouse	Collagen-induced arthritis	I.V.	Enhanced accumulation, reinforced therapeutic efficacy and minimal toxicity	[88]
Hybrid	Methotrexate in Folate Tagged Liposomes	Mouse	Collagen-induced arthritis	I.P.	Highly specific and efficient in targeting folate receptor β, and also significantly increase the clinical benefit	[107]
Hybrid	NF-κB decoy oligodeoxynucleotides, gold nanorods, and dexamethasone in folate modified liposomes	Mouse	Adjuvant-induced arthritis	I.V.	Significantly enhanced anti-inflammatory efficacy	[108]
Hybrid	NF-kB small interfering RNA and methothrexate in calcium phosphate/liposome	Mouse	Collagen-induced arthritis	I.V.	Effectively blocked the NF-kB signaling pathway, reduced the expression of proinflammatory cytokines	[90]
Hybrid	NF-kB targeted siRNA and methotrexate in a calcium phosphate/liposome-based hybrid nanocarrier	Mouse	Collagen-induced arthritis	I.D. *	Significant suppression of arthritis progression, targeting of macrophages, no decreased lymphocyte count	[90]
Hybrid	IL-27 liposomes coated with peptide ligand ART-1	Rat	Antigen-induced arthritis	I.V.	Better binding to endothelial cells, effective in suppressing disease progression, improved safety profile	[109]
Hybrid	Naringin, sulforaphane, and phenethyl isothiocyanate	Rat	Adjuvant-induced arthritis	I.V.	Antiarthritic activity observed after treatment with nutraceuticals	[92]
Hybrid	APO2L/TRAIL bound to liposomes	Rabbit	Adjuvant-induced arthritis	I.A.	Increased bioactivity compared to unmodified liposomes	[110]

* I.V.: intravenous, I.A.: intra-articular, PEG: polyethylene glycol, RDG: arginylglycylaspartic acid, DMARD: disease modifying antirheumatic drugs, I.P.: intraperitoneally, NABD: nucleic acid-based drug, I.D.: intradermally, S.C.: subcutaneously. All liposomal drugs were compared to the drug in free form, unless otherwise indicated.

**Table 2:** Overview of innovative research using liposome-based topical drug delivery in the treatment of psoriasis.

Route	Drug	Animal	Model	Challenge	Effect *	Ref.
Topical biologics	IL-17 Receptor targeting Liposomal Spherical Nucleic Acids vs. scrambled L-SNA	In vitro human skin	Healthy skin culture	Poor transdermal delivery	Reduced the expression of IL17RA by 72%	[38]
Topical biologics	IL-17 Receptor targeting Liposomal Spherical Nucleic Acids vs. scrambled L-SNA	Mouse	Imiquimod induced psoriatic plaque	Poor transdermal delivery	Reversed the development of psoriasis	[38]
Topical biologics	Liposomes containing plasmids with murine IL-4 gene	Mouse	K14-VEGF transgenic mice with moderate psoriasis	Topical transdermal gene delivery	Plasmid DNA was transdermally delivered and antipsoriatic efficacy was observed compared to untreated mice	[131]
Topical calcineurin inhibitor	Cyclosporine in liposomes vs. untreated animals	Mouse	Imiquimod induced psoriatic plaque	Adverse effects in systemic exposure, low topical absorption	Psoriatic features are markedly reduced after treatment	[123]
Topical calcineurin inhibitor	Cyclosporine in a liposomal formulation	Human	Psoriatic patients (*n* = 38)	Poor transdermal delivery	Treatment with cyclosporine lipogel resulted in complete clearance in 41% of psoriasis lesional sites in a safe manner; future efficacy studies are required	[124]
Topical keratolytic	Dipotassium glycyrrhizinate in elastic liposomes	Pig	Ex vivo porcine skin	Poor transdermal delivery	Elastic liposomes able to penetrate through membrane pores of diameter much smaller than their own diameter, and skin deposition increased 4.5-fold compared with aqueous solutions	[132]
Topical keratolytic	Dithranol in liposomes	Human	Psoriatic patients (*n* = 9)	Low stability and irritation in topical creams	5 patients were totally cleared of lesions, and a 50% reduction was achieved in two other patients	[130]
Topical keratolytic	Anthralin in liposomal and ethosomal gel	Human	Psoriatic patients	Reduction of side effects and increasing efficacy	No adverse effects were detected, and both formulations increased the efficacy of anthralin, with a significantly higher effect in ethosomes	[125]
Topical hybrid	all-trans retinoic acid and betamethasone in flexible liposomes	Mouse	Imiquimod induced psoriatic plaque	Enhanced therapeutic efficiency	Reduced thickness of epidermal layer and the level of proinflammatory cytokines compared to free drugs	[133]
Topical corticosteroid	Fusidic acid in liposomes	Mouse	Mouse tail model	Poor transdermal delivery	Increased permeation of the skin and increased efficacy	[134]
Topical corticosteroid	Methotrexate in oleic acid-containing deformable liposomes	Pig	Ex vivo porcine skin	Poor transdermal delivery	Liposomes with a size range of 80–140 nm showed enhanced skin permeability; inclusion of oleic acid increased deformability and enhanced permeability	[135]
Topical PUVA	Psoralen in liposomes and ethosomes	Rat	In vitro normal rat skin	Poor transdermal delivery	Transdermal flux and skin deposition using ethosomes were 3.50 and 2.15 times those achieved using liposomes, respectively	[136]
Topical methotrexate	Methotrexate in liposomes combined with laser targeting	Mouse	Healthy skin	Systemic treatment is limited due to several adverse effects	Treated mice showed no recurrence of psoriasis symptoms	[129]
Topical vitamin D analogue	Calcipotriol in PEGylated liposomes	Pig	Ex vivo porcine skin	Poor transdermal delivery	Liposome size affects penetration into the stratum corneum; deposition improved slightly with PEGylated liposomes vs. unPEGylated liposomes	[137]

* All liposomal drugs were compared to the drug in free form, unless otherwise indicated.

**Table 3:** Overview of innovative research using liposome-based drug delivery in the treatment of vasculitis, atherosclerosis, and arteriovenous fistulas.

Goal	Liposome Formulation	Animal	Model	Effect *	Ref.
Cholesterol entrapment	Synthetic dimyristoylphosphatidylcholine liposomes in high-density lipoprotein	Rabbit	Cholesterol-fed rabbits	Significantly decreased aortic cholesterol contents and decreased artherosclerotic plaque volume compared to untreated animals	[145]
Vaccination—Activation of atheroprotective peritoneal B1a cells	Phosphatidylserine liposomes vs. control liposomes	Mouse	ApoE-KO mice	Reduction of oxidized LDL in the lesion and reduced necrotic core size	[157]
Artherosclerosis vaccine	Anionic 1,2-distearoyl-sn-glycero-3-phosphoglycerol liposomes	Mouse	Western type diet	Induction of antigen-specific Tregs, reduced plaque formation, increased plaque stability	[158]
Vaccination—inducing anticholesterol IgG and IgM antibodies	Liposomes containing 71% cholesterol and lipid A as an adjuvant	Rabbit	A diet containing 0.5% to 1.0% cholesterol	Decrease in elevation of serum cholesterol accompanied by reduced antibody levels, indicating antibody mediated decrease; also: decreased artherosclerosis risk and decreased plaque size compared to nonimmunized animals	[146]
Vaccination—inducing anticholesterol antibodies	Cholesterol liposomes	Rabbit	High cholesterol diet	Immunization was effective in preventing artherosclerotic plaque formation compared to negative control animals, but this effect was absent upon immunostimulation with a Gram-negative bacterial product	[159]
Induction of tolerance in dendritic cells and T cells	Liposomes encapsulating calcitriol, and PD-L1	In vitro and mice	Goodpasture’s vasculitis model	In vitro induction of Tregs was observed, and the severity of vasculitis was reduced in vivo compared to untreated animals	[142]
Evaluate echogenic liposome delivery	Rhodamine-labeled echogenic liposomes	Mouse	Ex vivo aortae from ApoE-deficient mice	Subendothelial delivery of rhodamine liposomes was observed in ultrasound treated aortae but not in untreated samples; no ultrasound-mediated damage was observed	[160]
Stem cell delivery and ultrasound guided release	CD34 and ICAM-1 coupled echogenic immunoliposomes	Pig	High cholesterol diet	Stem cells were successfully delivered to the arterial intima, and this effect was enhanced upon ultrasound treatment	[151]
Delivery of liposomal si-RNA	Fatty Acid Binding Protein 4 si-RNA in liposomes vs. control si-RNA in liposomes	Mouse	ApoE-deficient mice	Successful delivery of siRNA to artherosclerotic plaques and successful suppression of FABP4 expression	[150]
Drug delivery	Statins in a reconstituted high-density lipoprotein nanoparticle carrier	Mouse	ApoE-deficient mice	Inhibition of the inflammatory progression within atherosclerotic plaques	[149]
Drug delivery	NADPH oxidase inhibitor in immunoliposomes targeted to endothelial marker platelet endothelial cell adhesion molecule	Mouse	LPS challenged lungs	Alleviation of pathological disruption of endothelial permeability barrier function compared to untreated animals	[161]
Drug delivery	Prednisolone phosphate in PEGylated liposomes	Human	Patients receiving an arteriovenous fistula in the forearm	The clinical trial was concluded prematurely due to low inclusion, and no treatment effect was observed	[162]
Drug delivery	Prednisolone in liposomes	Mouse	Western type diet	Atherosclerosis was accelerated with increased macrophage content, larger necrotic cores, and more advanced plaque formation compared to empty liposome treatment	[163]
Drug delivery	anti-VCAM-1 and anti-E-selectin short interferin RNAs in cationic amphiphile SAINT-C18 liposomes	Human cells and mouse	Human umbilical vein endothelial cells and human aortic endothelial cells, and TNF-α induced mice	Successful delivery to in vitro cultured endothelial cells and subsequent downregulation of target mRNA; in vivo pharmacokinetics were comparable to conventional PEGylated liposomes	[164]
Drug delivery	Dexamethasone in liposomes	Mouse	Aortic artherosclerotic lesions	Liposomes of 202 nm diameter had optimal uptake in aortic lesions, allowing for lower dose treatment	[165]
Drug delivery to circulating monocytes	Liposomal Alendronate vs. placebo	Human	Patients undergoing bare metal stent implantation	Intravenous administration is safe and effectively modulates monocyte behavior	[166]
Drug delivery to atherosclerotic plaque macrophages	Prednisolone in liposomes vs. placebo	Human	Healthy volunteers and patients with atherosclerotic disease	Intravenous injection with liposomal prednisolone led to accumulation in plaque macrophages, but anti-inflammatory efficacy was not observed	[148]
Drug delivery	Prednisolone phosphate in PEGylated liposomes	Rabbit	Artherosclerotic plaques induced by high cholesterol diet	Local accumulation in the plaque was achieved and prednisolone treatment was efficient	[147]
Drug delivery	Fumagillin-loaded liposomes compared to empty liposomes	Mouse	Apolipoprotein E-knockout (ApoE-KO) mice	Decrease in lesion size	[167]

* All liposomal drugs were compared to the drug in free form, unless otherwise indicated.

**Table 4:** Overview of innovative research using liposome-based drug delivery in organ transplantation.

Goal	Liposome Formulation	Animal	Model	Effect *	Ref.
Gene therapy delivery in heart transplantation	IL-10 gene plasmid in liposomes	Rat	Functional heterotopic heart transplantation	Gene transfer efficiency was lower than the adenovirus group, but the efficacy of liposome mediated transfer was higher	[187]
Gene therapy in lung transplantation	Hemagglutinating virus of Japan gene transfer system containing plasmid DNA in liposomes	Rat	Organ perfusion or intratracheal instillation of liposomes during lung transplantation	Low levels of gene transfer to endothelial cells, and moderate transfection to airway and alveolar cells	[189]
Drug delivery during chronic rejection	Chlodronate liposomes	Rat	Chronic allograft rejection	Reduced expression of proinflammatory cytokines and reduced T cell proliferation compared to untreated animals	[190]
Drug delivery during acute rejection	Prednisolone in PEGylated liposomes	Mouse	Acute cellular rejection after kidney transplantation	Liposomes accumulated in the transplanted kidney and liposomal prednisolone improved the efficacy in attenuating the renal inflammation	[186]
Drug delivery on the organ level	Prednisolone in PEGylated liposomes	Rat	Renal ischemia reperfusion damage	Liposomes accumulate locally in the inflamed kidney and seem to extravasate via peritubular capillaries	[56]
Drug delivery after lung transplantation	Cyclosporin in liposomes	Human	Lung transplant patients suffering from bronchiolitis obliterans syndrome (BOS)	An increased efficacy on BOS free survival was observed, compared to oral cyclosporin, with no systemic toxicity	[188]
Drug delivery during cell graft	Liposomal tacrolimus and rapamycin	Rat	Fetal ventral mesencephalic cell transplantation into the brains of rats with unilateral 6-hydroxydopamine lesions	Higher survival of cell grafts and increased fiber outgrowth after synergistic treatment using tacrolimus and rapamycin compared to separate administration	[184]
Drug delivery during xenotransplantation	Tacrolimus in liposomes	Rat	Xenotransplantation of mouse cells into a hemiparkinsonian rat	Increased survival of xenotransplanted cells and a decrease in rotational behavior compared to untreated animals	[191]

* All liposomal drugs were compared to the drug in free form, unless otherwise indicated.
